# ^18^FDG‐avid cholesterol granuloma mimicking residual aggressive lymphoma after chemotherapy

**DOI:** 10.1002/jha2.332

**Published:** 2021-11-09

**Authors:** Lorenzo Falchi, Filiz Sen

**Affiliations:** ^1^ Department of Medicine, Lymphoma Service Memorial Sloan‐Kettering Cancer Center New York New York USA; ^2^ Department of Pathology, Hematopathology Service Memorial Sloan‐Kettering Cancer Center New York New York USA

A 54‐year‐old man presented with abdominal pain, drenching night sweats, weight loss and left inguinal lymphadenopathy, which was biopsy‐proven to be follicular lymphoma with transformation to diffuse large B‐cell lymphoma. A staging ^18^F‐fluorodeoxyglucose‐positron emission tomography (FDG‐PET) revealed extensive, metabolically active lymphadenopathy, pleural and peritoneal nodules, and T12 involvement (Figure 1A).

**FIGURE 1:**
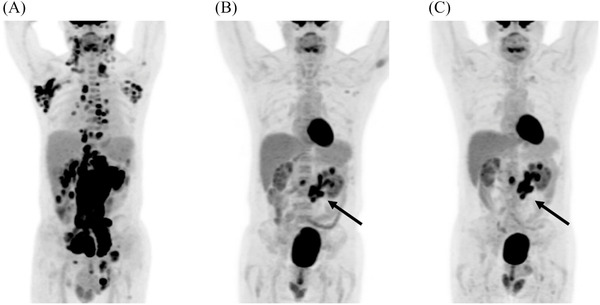
Serial select positron emission tomography‐computed tomography (PET‐CT) images during treatment: (A) before R‐CHOP chemoimmunotherapy initiation, (B) at the end of six cycles of R‐CHOP, and (C) 6 weeks after completion of R‐CHOP

The patient received rituximab‐cyclophosphamide‐doxorubicin‐vincristine‐prednisone for six cycles. The end‐of‐treatment PET showed increased ^18^F‐FDG‐avid mesenteric nodes and densities (Figure 1B, arrow), which persisted at a 6‐week follow‐up (Figure 1C, arrow).

Pathologic examination of mesenteric lesions revealed fibroadipose tissue and a nodular histiocytic proliferation with cholesterol clefts, consistent with cholesterol granuloma (CG), without evidence of lymphoma, mycobacteria, or fungi (Figure 2A,B). The histiocytes expressed CD68, CD163 (Figure 2C), CD11c, CD33, and PU.1 and were negative for PAX5 (Figure 2D), CD19 (Figure 2E), CD23, CD1a, S100, ALK, and BRAF.

**FIGURE 2:**
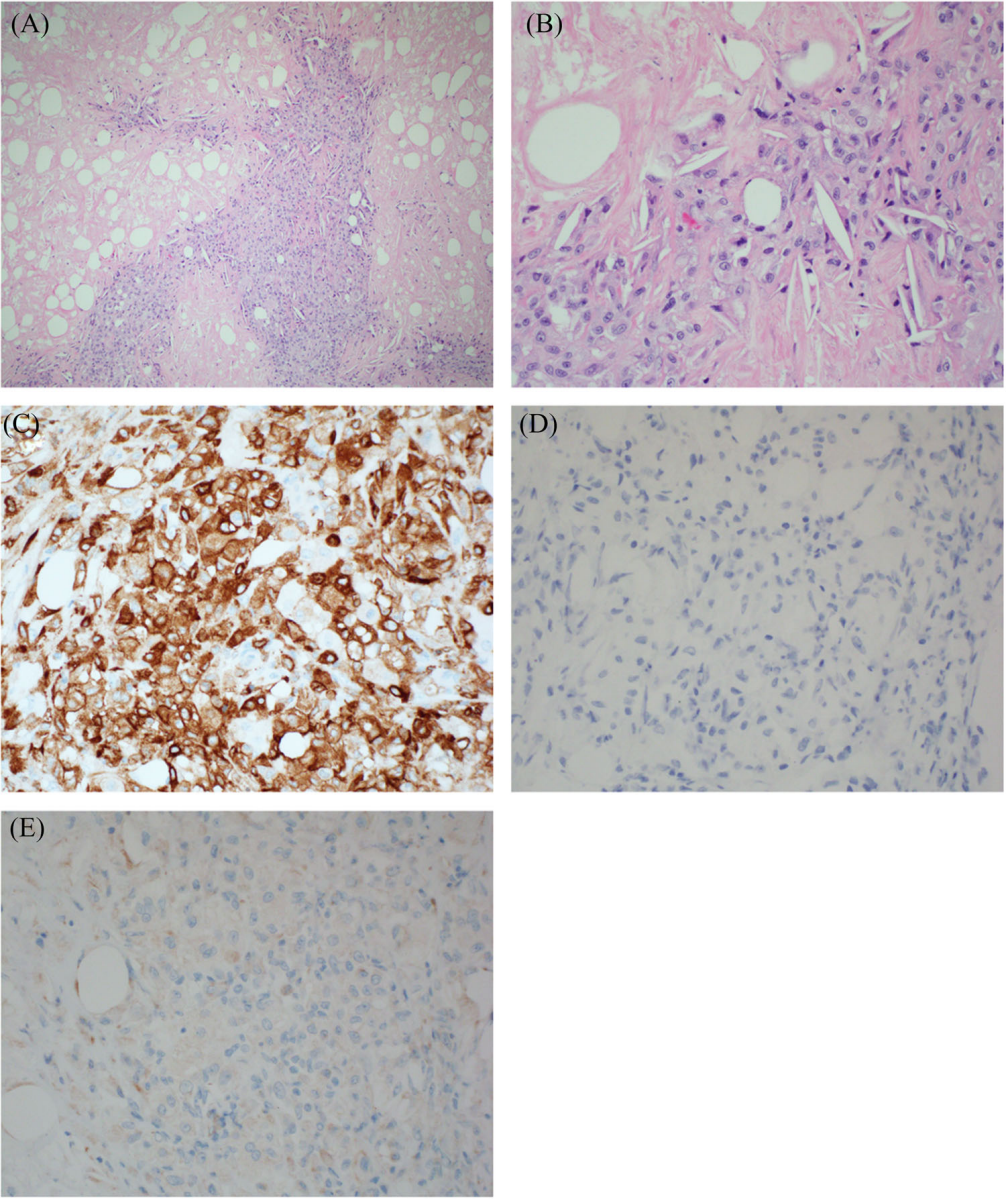
Microphotograph of retroperitoneal lymph node core needle biopsy specimen (A,B) Hematoxylin and eosin (H&E) stain, (C) the histiocytic proliferation is positive for CD163 (D) immunostain for for PAX5 is negative. (E) immunostain for CD19 is negative.

The pathogenesis of CG remains unclear. It is believed that local hemorrhage, resulting from trauma, inflammation, neoplasia, or tumor necrosis (as in our patient) causes degeneration of cells, which develops cholesterol crystals. These may induce a foreign‐body‐type giant‐cell reaction leading to granuloma formation. As ^18^F‐FDG accumulation can be observed at CG sites, our case highlights CG as a potential pitfall in interpreting ^18^F‐FDG‐PET results after lymphoma therapy.

## AUTHOR CONTRIBUTIONS

Lorenzo Falchi provided medical care to the patient and selected the radiology images. Filiz Sen analyzed all pathology specimens and took the pictures. Both Lorenzo Falchi and Filiz Sen designed the research, analyzed the data, wrote and approved the manuscript.

## CONFLICT OF INTEREST

The authors declare that they have no conflict of interest.

